# In the national epidemiological bulletins – a selection from recent issues

**DOI:** 10.2807/1560-7917.ES.2019.24.26.1906271

**Published:** 2019-06-27

**Authors:** Eurosurveillance editorial team

**Affiliations:** 1 European Centre for Disease Prevention and Control (ECDC), Stockholm, Sweden

**Keywords:** infectious diseases, public health, Europe

**Healthcare-associated infections and antibiotic resistance**

 

Point prevalence survey of hospital-acquired infections and antimicrobial use in Irish hospitals

Epi-Insight 2019;20(5)

May 2019, Ireland

http://ndsc.newsweaver.ie/epiinsight/1djgbqmwsby?a=1&p=54935686&t=17517774

 

MRSA in Saxony-Anhalt

Epidemiologisches Bulletin 13/2019

28 March 2019, Germany

https://www.rki.de/DE/Content/Infekt/EpidBull/Archiv/2019/Ausgaben/13_19.pdf?__blob=publicationFile

 

**Food- and waterborne diseases**

 

Cryptosporidiosis outbreak within a middle school in western France, November 2017

Bulletin épidémiologique hebdomadaire, 16/2019

11 June 2019, France

http://invs.santepubliquefrance.fr/beh/2019/16/pdf/2019_16.pdf

 

Ongoing outbreak with *Yersinia* infection

EPI-NEWS 18, 2019

27 May 2019, Denmark

https://en.ssi.dk/news/epi-news/2019/no-18---2019

 

Shiga toxin-producing *Escherichia coli* (STEC) and haemolytic uraemic syndrome (HUS) in the 2014-2018 period

EPI-NEWS 18, 2019

27 May 2019, Denmark

https://en.ssi.dk/news/epi-news/2019/no-18---2019

 

Listeria trends and monitoring in Denmark, 2014-2018

EPI-NEWS 18, 2019

27 May 2019, Denmark

https://en.ssi.dk/news/epi-news/2019/no-18---2019

 

Rotavirus notifications declined sharply in 2018

Epi-Insight 2019;20(5)

May 2019, Ireland

http://ndsc.newsweaver.ie/epiinsight/1musqleykke?a=1&p=54935683&t=17517774

 

**Hepatitides**

 

Dates as the likely vehicle in a hepatitis A outbreak among travellers returning from Morocco, 2018

Epidemiologisches Bulletin 25/2019

20 June 2019, Germany

https://www.rki.de/DE/Content/Infekt/EpidBull/Archiv/2019/Ausgaben/25_19.pdf?__blob=publicationFile

 

Epidemiology of hepatitis C, 2018

Epi-Insight 2019;20(6)

June 2019, Ireland

http://ndsc.newsweaver.ie/epiinsight/16uf7b81d7o?a=1&p=55108367&t=17517774

 

Laboratory reports of hepatitis A infections in England and Wales, October to December 2018 

Health Protection Report; 13(14)

26 April 2019, United Kingdom

https://assets.publishing.service.gov.uk/government/uploads/system/uploads/attachment_data/file/797710/hpr1419_hepA.pdf

 

**Vaccine-preventable diseases**

 

HAIBA 2018 - Study of the vaccination coverage for the 5-year booster in Copenhagen

EPI-NEWS 20, 2019

25 June 2019, Denmark

https://en.ssi.dk/news/epi-news/2019/no-20---2019

 

Laboratory confirmed cases of measles, rubella and mumps, England: January to March 2019

Health Protection Report; 13(18)

24 May 2019, United Kingdom

https://assets.publishing.service.gov.uk/government/uploads/system/uploads/attachment_data/file/804024/hpr1819_mmr2.pdf

 

Transmission of cutaneous diphtheria within a family – first diphtheria outbreak in Germany in almost 40 years

Epidemiologisches Bulletin 20/2019

16 May 2019, Germany

https://www.rki.de/DE/Content/Infekt/EpidBull/Archiv/2019/Ausgaben/20_19.pdf?__blob=publicationFile

 

Laboratory-confirmed whooping cough 2018

EPI-NEWS 15, 2019

15 May 2019, Denmark

https://en.ssi.dk/news/epi-news/2019/no-15---2019

 

Laboratory confirmed cases of pertussis (England): annual report for 2018

Health Protection Report; 13(14)

26 April 2019, United Kingdom

https://assets.publishing.service.gov.uk/government/uploads/system/uploads/attachment_data/file/797712/hpr1419_prtsss-ann.pdf

 

Cumulative shingles vaccine coverage report to end of March 2019: England

Health Protection Report; 13(14)

26 April 2019, United Kingdom

https://assets.publishing.service.gov.uk/government/uploads/system/uploads/attachment_data/file/797713/hpr1419_shngls_vc.pdf

 

Pertussis vaccination programme for pregnant women update: vaccine coverage in England, October to December 2018

Health Protection Report; 13(14)

26 April 2019, United Kingdom

https://assets.publishing.service.gov.uk/government/uploads/system/uploads/attachment_data/file/797814/HPR1419_prntl-prtsss_vaccine_coverage_October_to_December_2018.pdf

 

Measles epidemiology in France between 2011 and 2018

Bulletin épidémiologique hebdomadaire, 13/2019

23 April 2019, France

http://invs.santepubliquefrance.fr/beh/2019/13/pdf/2019_13.pdf

 

Mumps outbreak in Ireland, 2019

Epi-Insight 2019;20(4)

April 2019, Ireland

http://ndsc.newsweaver.ie/epiinsight/amp6s59eba810gkzp9yxn5?a=1&p=54794825&t=17517774

 

Estimation of vaccination coverage in adolescents from Ille-et-Vilaine district: results of repeated cross-sectional survey based on the Defense and Citizenship Day in Rennes, France, 2015-2018

Bulletin épidémiologique hebdomadaire, 14/2019

7 May 2019, France

http://invs.santepubliquefrance.fr/beh/2019/14/pdf/2019_14.pdf

 

**Respiratory diseases**

 

Legionnaires’ disease 2018

EPI-NEWS 19, 2019

20 June 2019, Denmark

https://en.ssi.dk/news/epi-news/2019/no-19---2019

 

Legionnaires’ disease outbreak in the north of Ghent

Newsflash Infectious Diseases

June 2019, Brussels

https://epidemio.wiv-isp.be/ID/Report/Juin_2019.pdf

 

Human adenovirus infections

Epidemiologisches Bulletin 22/2019

29 May 2019, Germany

https://www.rki.de/DE/Content/Infekt/EpidBull/Archiv/2019/Ausgaben/22_19.pdf?__blob=publicationFile

 

Epidemiology of tuberculosis in Ile-de-France: An increase of cases reported in 2016 and 2017

Bulletin épidémiologique hebdomadaire, 14/2019

7 May 2019, France

http://invs.santepubliquefrance.fr/beh/2019/14/pdf/2019_14.pdf

 

**Sexually transmitted diseases**

 

Sexually transmitted infections and screening for chlamydia in England, 2018

Health Protection Report; 13(19)

7 June 2019, United Kingdom

https://assets.publishing.service.gov.uk/government/uploads/system/uploads/attachment_data/file/806118/hpr1919_stis-ncsp_ann18.pdf

 

Diagnoses of STIs continue to rise in Scotland

HPS Weekly Report; 53(21)

28 May 2019, Scotland

https://www.hps.scot.nhs.uk/publications/hps-weekly-report/volume-53/issue-21/diagnoses-of-stis-continue-to-rise-in-scotland/

 

STIs in young people in Ireland

Epi-Insight 2019;20(5)

May 2019, Ireland

http://ndsc.newsweaver.ie/epiinsight/14zi7yv8oji?a=1&p=54935685&t=17517774

 

HIV drug resistance in Ireland - improving prevalence estimates

Epi-Insight 2019;20(5)

May 2019, Ireland

http://ndsc.newsweaver.ie/epiinsight/17d8xtlu156?a=1&p=54935679&t=17517774

 

**Zoonoses and vector-borne diseases**

 

Common animal associated infections quarterly report (England and Wales): first quarter 2019

Health Protection Report; 13(20)

14 June 2019, United Kingdom

https://assets.publishing.service.gov.uk/government/uploads/system/uploads/attachment_data/file/809141/hpr2019_zoosQ1.pdf

 

Lyme disease: raising awareness

Epi-Insight 2019;20(6)

June 2019, Ireland

http://ndsc.newsweaver.ie/epiinsight/lhp3kto4dli?a=1&p=55108364&t=17517774

 

Development of management guidelines for the management of Lyme disease: an ethical dimension is needed

Bulletin épidémiologique hebdomadaire, 14/2019

7 May 2019, France

http://invs.santepubliquefrance.fr/beh/2019/14/pdf/2019_14.pdf

 

The holistic approach of patients consulting for suspected Lyme borreliosis results in 9.6% diagnostic confirmation

Bulletin épidémiologique hebdomadaire, 14/2019

7 May 2019, France

http://invs.santepubliquefrance.fr/beh/2019/14/pdf/2019_14.pdf

 

TBE detected on Funen and in Jutland in 2018

EPI-NEWS 12-14, 2019

10 April 2019, Denmark

https://en.ssi.dk/news/epi-news/2019/no-12-14---2019

 

**Other**

 

Screening of pregnant women for hepatitis B, HIV and syphilis, 2018

EPI-NEWS 21, 2019

25 June 2019, Denmark

https://en.ssi.dk/news/epi-news/2019/no-21---2019

 

Blood donor screening 2018

EPI-NEWS 19, 2019

20 June 2019, Denmark

https://en.ssi.dk/news/epi-news/2019/no-19---2019

 

A norovirus outbreak in an outdoors playground: One-Health approach

Infectieziekten Bulletin 2019; 30(5)

May–June 2019, the Netherlands

https://magazines.rivm.nl/2019/05/infectieziekten-bulletin/een-norovirusuitbraak-een-natuurspeeltuin-een-one-health-benadering

 

Special edition: Health recommendations for travellers, 2019 (for health professionals)

Bulletin épidémiologique hebdomadaire

21 May 2019, France

http://invs.santepubliquefrance.fr/Publications-et-outils/BEH-Bulletin-epidemiologique-hebdomadaire/Archives/2019/BEH-hors-serie-Recommandations-sanitaires-pour-les-voyageurs-2019

 

Group A streptococcal infections: third report on seasonal activity, 2018 to 2019

Health Protection Report; 13(16)

10 May 2019, United Kingdom

https://assets.publishing.service.gov.uk/government/uploads/system/uploads/attachment_data/file/800932/hpr1619_gas-sf3.pdf

